# Arterial hypertension in Leigh syndrome due to m.13513G > A is multicausal, requiring an extensive search to identify its pathphysiology

**DOI:** 10.1186/s40885-023-00247-4

**Published:** 2023-07-01

**Authors:** Josef Finsterer

**Affiliations:** Neurology and Neurophysiology Center, Postfach 20, Vienna, 1180 Austria

**Keywords:** mtDNA, Arterial hypertension, Leigh syndrome, Catecholamines, Brain, Cardiac

## Letter to the editor

We read with interest the article by Solis et al. on a 3 years and 10 months-old female with Leigh syndrome due to the variant m.13513G > A in *MT-ND5* with a heteroplasmy rate of 69% [1]. Leigh syndrome phenotypically manifested with developmental delay, hypotonia, failure to thrive, lactic acidosis, multiple, bilaterally symmetric DWI hyperintensities in the sub-thalamic nuclei, brainstem, and upper cervical spine, strabismus, Wolff-Parkinson-White (WPW) syndrome, obstructive sleep apnoea, hypo-gamma-globulinemia, proximal tubular acidosis, and renal cysts [1]. By age 3y, malignant arterial hypertension associated with increased catecholamines developed, but extensive investigations were inconclusive as to why the hypertensive crises were attributed to catecholamine-induced autonomic dysfunction [1]. The study is excellent but has limitations that should be discussed.

The main limitation of the study is that multiple causes of arterial hypertension were not adequately ruled out. Arterial hypertension is a well-known phenotypic feature of mitochondrial disorders (MIDs) and has been reported particularly in Chinese MID patients [2]. Therefore, it is conceivable that arterial hypertension was simply a phenotypic manifestation of the mtDNA mutation. An argument for this speculation is that arterial hypertension has previously been reported in m.13513G > A carriers [2]. In a 12 months-old infant, the m.13513G > A variant manifested with tachy-arrhythmias, hyponatriemia, inappropriate ADH secretion, cardiomyopathy, WPW-syndrome, and arterial hypertension [3].

There is also evidence that MIDs manifest with neoplasms more commonly than non-MIDs. Therefore, we should know whether catecholamine-producing neoplasms, benign or malign, have been excluded as the cause of hypertension in the index patient. In particular, extra-suprarenal pheochromocytoma or paraganglioma must be ruled out. The 18 F-FDOPA PET/CT can be helpful in this respect but a negative result does not rule out paraganglioma [4].

Another unaccounted cause of arterial hypertension is an ACTH-producing micro- or macro-adenoma of the pituitary gland. Pituitary adenoma can be a phenotypic manifestation of MIDs, making it crucial that such neoplasms are adequately ruled out. However, serum cortisol levels were not determined and no dexamethasone suppression test was performed. A negative dexamethasone suppression test does not rule out non-ACTH producing pituitary adenoma.

Another cause of arterial hypertension in patients with Leigh syndrome is increased intra-cranial pressure [5]. Therefore, an ophthalmologic examination to rule out papilledema or a lumbar puncture and direct measurement of the intra-cranial pressure is mandatory.

It is also conceivable that arterial hypertension resulted from unwitnessed generalised seizures or non-convulsive status epilepticus. Since Leigh syndrome is often associated with seizures, we should know if epileptiform discharges were recorded on EEG.

We disagree with the classification of DWI hyperintensities as ischemic stroke [1]. Ischemic stroke is characterised by DWI hyperintensity and hypointensity on apparent diffusion coefficient (ADC) maps (cytotoxic edema). However, no ADC maps have been described or shown in a figure. Another argument against ischemic stroke is the symmetry of the lesions. These lesions are typically of Leigh syndrome but atypically of ischemic stroke. A third argument against ischemic stroke is that the lesions were small and not confined to a vascular territory.

There is no mention of the family history, particularly whether the index patient’s mother also carried the *MT-ND5* variant and whether she manifested phenotypically with arterial hypertension. mtDNA variants are transmitted via the maternal line in 75% of cases [6]. Therefore, it is necessary not only to examine the mother clinically but also to test her genetically.

Overall, the interesting study has limitations that call the results and their interpretation into question. Clarifying these weaknesses would strengthen the conclusions and could improve the study. Arterial hypertension in Leigh syndrome can be multicausal, thus requiring comprehensive workup to identify the underlying pathphysiology.

## Data Availability

All data used are available from the corresponding author.
